# 25-Hydroxyvitamin D Serum Levels Linked to Single Nucleotide Polymorphisms (SNPs) (rs2228570, rs2282679, rs10741657) in Skeletal Muscle Aging in Institutionalized Elderly Men Not Supplemented with Vitamin D

**DOI:** 10.3390/ijms231911846

**Published:** 2022-10-06

**Authors:** Diego Fernández-Lázaro, Juan Luis García Hernández, Eva Lumbreras, Juan Mielgo-Ayuso, Jesús Seco-Calvo

**Affiliations:** 1 Department of Cellular Biology, Genetics, Histology and Pharmacology, Faculty of Health Sciences, Campus of Soria, University of Valladolid, 42003 Soria, Spain; 2 Neurobiology Research Group, Faculty of Medicine, University of Valladolid, 47005 Valladolid, Spain; 3 Molecular Mechanisms of Cancer Program, Institute of Molecular and Cellular Biology of Cancer, Spanish National Research Council (CSIC), University of Salamanca, 37007 Salamanca, Spain; 4 Institute of Biomedical Research of Salamanca (IBSAL), Department of Hematology, Salamanca University Hospital, 37007 Salamanca, Spain; 5 Institute of Biomedical Research of Salamanca (IBSAL), Cytogenetics-Molecular Genetics in Onco-Hematology, Cancer Research Center-University of Salamanca (IBMCC, USAL-CSIC), 37008 Salamanca, Spain; 6 Department of Health Sciences, Faculty of Health Sciences, University of Burgos, 09001 Burgos, Spain; 7 Physiotherapy Department, Institute of Biomedicine (IBIOMED), Campus of Vegazana, University of Leon, 24071 Leon, Spain; 8 Physiology Department, Faculty of Medicine, Basque Country University, 48900 Leioa, Spain

**Keywords:** elderly, aging, sarcopenia, 25-OH vitamin D, deficiency, genetics, CYP2R1, GC, VDR

## Abstract

Sarcopenia (Sp) is the loss of skeletal muscle mass associated with aging that results in an involution of muscle function and strength. Vitamin D deficiency is a common health problem worldwide, especially among the elderly, and hypovitaminosis D leads to musculoskeletal disorders. The aim of this study was to evaluate the impact and presence of a possible linkage between Single Nucleotide Polymorphisms (SNPs) CYP2R1 (rs10741657), GC (rs2282679), and VDR (rs2228570), serum 25-OH/D concentrations and the link with the degree of sarcopenia in 19 institutionalized elderly men not supplemented with vitamin D. Levels of 25-OH vitamin D were quantified with a commercial enzyme-linked immunosorbent assay kit and 3 SNPs were genotyped with KASPar assays. Significant differences in 25-OH/D concentration were determined between the bi-allelic combinations of rs228679 and rs228570. We detected statistically significant weak positive correlations between the AA (rs10741657 and rs228570) and TT (rs228679) and alleles and 25-OH/D and the probability of having higher 25-OH/D concentrations was 2- to 3-fold higher. However, the GG alleles of the 3 SNPs showed that the probability of having optimal 25-0H/D concentrations decreases by 32% for rs10741657, 38% for rs228679, and 74% for rs228570, showing a strong negative correlation between the degree of sarcopenia and 25-OH/D levels. Allelic variations in CYP2R1 (rs10741657), GC (rs2282679), and VDR (rs10741657) affect vitamin D levels and decisively influence the degree of sarcopenia in institutionalized elderly people.

## 1. Introduction

One of the most relevant physiological changes of aging, due to its dramatic consequences, is the loss of skeletal muscle mass and function [1]. This process of muscle tissue involution is known as “*Sarcopenia*” [2]. Sarcopenia, according to the European Working Group on Sarcopenia in Older People (EWGSOP), is defined as a progressive and generalized disorder of skeletal muscle and is categorized according to losses in muscle strength, physical performance, and quantity/quality of skeletal muscle mass [3].

Vitamin D is considered an essential fat-soluble steroid hormone involved in a wide variety of physiological processes, suggesting that maintaining adequate vitamin D levels may benefit health outcomes [4]. Vitamin D is crucial for maintaining musculoskeletal health because it regulates immune function, inflammatory response, synthesis of proteins that promote cell growth, and modulates skeletal muscle contractile function [5]. Therefore, one of the main target tissues of vitamin D is the skeletal muscle [6], and hypovitaminosis D would cause musculoskeletal disorders such as muscle weakness, myopathy, and altered proliferation and differentiation of muscle fibers [7]. These disorders would directly influence a loss of muscle strength and a decrease in physical performance [8]. Vitamin D deficiency is a common health problem worldwide, particularly among the elderly [9]. Several factors are involved in changes in vitamin D status in aging such as the reduced capacity for its synthesis in the skin pigmentation, adiposity, use of medications, reduced renal function, low dietary vitamin D intake, and particularly in institutionalized elderly people, low exposure to sunlight because they spend more time indoors [10,11,12]. However, only a portion of the interindividual variability of 25-hydroxyvitamin D (25-OH/D) is attributable to these causes. Previous studies reported that genetic factors contribute significantly to the variability of 25-OH/D with heritability estimates >50% [13,14]. Lower serum 25-OH/D concentrations are associated with greater risks of many chronic diseases [15]. Therefore, older people with genotype-associated vitamin D deficiency may be at risk for sarcopenia [16].

However, there are no studies that relate genetic modifiers of vitamin D status to the degree of sarcopenia; but several functions of this vitamin have been described that are associated with adequate muscle health [5]. Thus, genetic determinants of circulating 25-OH/D may influence muscle status thus influencing the variables that determine the diagnosis of sarcopenia. Several genes underlie the serum concentration of 25-OH/D by influencing metabolism and transport. Cytochrome P450 2R1 (CYP2R1) codes for 25-hydroxylase vitamin D, an enzyme that converts vitamin D to 25-OH/D, the main circulating form of vitamin D [17]. The group-specific component (GC), also known as vitamin D-binding protein (DBP), encodes a protein belonging to the albumin family that binds vitamin D and transports it to target tissues [13,18]. Various studies have reported that genetic mutations in these genes have been associated with vitamin D deficiency [13,14,18,19]. Additionally, the VDR gene encodes for the receptor that binds to activated vitamin D3 and regulates many biological activities including calcium and phosphorous homeostasis, apoptosis, and cell differentiation [20]. VDR genes have been linked to vitamin D deficiency [15,21]. Three common single nucleotide polymorphisms (SNPs) rs10741657 to CYP2R1 (allelic variants A > G), rs2282679 to GC (allelic variants T > G) and rs2228570 to VDR (allelic variants A > G) influence vitamin D status. Biallelic variants of these SNPs generate different gene products/protein isoforms that influence the functional effect on proteins: CYP2R1 (rs10741657) differ in the working enzymatic activity of 25-hydroxylase; GC (rs2282679) differ in their vitamin D binding/transport affinity; VDR (rs2228570) differ in the functional uptake of vitamin D receptor. SNPs had a function already described in the literature based on their previous association with 25-OH/D and its related genotypes [13,18,21,22,23]. Thus, depending on the nature of the polymorphism, it could either increase or decrease the concentration of 25-OH/D in sera.

Against this background, we set out to conduct a study on institutionalized elderly, to evaluate the impact and presence of a possible connection between CYP2R1 (rs10741657), GC (rs2282679), and VDR (rs2228570), serum 25-OH/D concentrations and the link with the degree of sarcopenia in older adults. Knowing these relationships could be of great interest to establish the links between the alleles of each SNPs and the levels of Vitamin D associated with the health status of individuals, favoring the development of individualized strategies for Vitamin D supplementation.

## 2. Results

### 2.1. Sample and Lifestyle Characteristics

The sample consisted of 19 men (100%), older adults were 82.9 ± 6.7 years; most participants being born in Spain (89.5%). Regarding lifestyle-related characteristics, the mean BMI of 27.2 ± 5.2 kg/m^2^ evidenced that the sample was overweight according to SEEDO criteria [24], SMMI was 9.1 ± 2.6 kg/m^2^, sun exposure was 17.37 ± 6.76 min per day and 14 subjects were exposed to the sun >15 min per day. A total of 52.6% were non-smokers. The proportion of adherent patients to the Mediterranean diet according to the Trichopoulou et al. [25] questionnaire was 10.4 ± 1.9. The total sample reported 72.9 ± 14.7 of self-perceived well-being, as assessed by the Visual Analogue Scale (VAS) adapted by Gould et al. [26] (Table 1).

### 2.2. Physical Fitness Condition

For institutionalized patients, the handgrip strength was 26.4 ± 5.6 kg/cm^2^ and 19.6 ± 4.3 kg/cm^2^ in the dominant hand and the non-dominant hand, respectively. Likewise, 26.3% of the institutionalized patients were below the specific cut-off point, in physical performance measures (“*Get Up and Go Test*”), which identifies and characterizes sarcopenia according to EWGSOP [30] (Table 1).

### 2.3. Clinical Description

None of the 19 study participants were infected by SARS-CoV-2 before. Only 1 institutionalized patient used oxygen therapy and 26.6% of the participants suffered allergies. Arterial hypertension (89.9%) were the most chronic conditions among study participants, followed by cardiovascular (73.6%), insulin-dependent diabetes mellitus (10.5%), respiratory disease (10.5%), obesity (5.2%), and cancer (5.2%). The usual pharmacological treatments used by the study participants were mostly antihypertensives (89.9%) followed by anxiolytics/sedatives (84.2%), cardiovascular drugs (73.6%), anticoagulants (15.7%), lipid-lowering agents (15.7%), antidiabetics (10.5%), and immunosuppressants (5.2%). Concerning vital signs were SBP 127 ± 14.0 mmHg, DBT 72.3 ± 12.4 mmHg, Heart rate 76.1 ± 12.7 bpm, temperature 35.8 ± 0.5 °C, and oxygen saturation 98.9 ±1.9% (Table 1).

### 2.4. 25-OH/D Level

25-OH/D level of the total institutionalized elderly was 22.7 ± 10.1 ng/mL. The 25-OH/D levels for participants < 70 years was 26.5 ± 11.8 ng/mL and in those >70 years was 19.5 ± 7.8 ng/mL (Table 2). A total of 68.4% of the elderly had 25-OH/D level insufficiency. In participants <70 years, 50% were in the range of insufficiency and 37.5% in normal values. In the elderly >70 years, 81.8% had 25-OH/D level insufficiency (Table 2). According to SEIOMM guidelines [31], the cohort examined belongs to the population at risk of osteoporosis.

### 2.5. Sarcopenia Degrees

Ten patients were identified with sarcopenia according to EWOPSOP criteria [30]. For rs10741657 (CYP2R1) 1 subject for AA, 4 for GA, and 5 for GG were sarcopenic patients. Five older adults had sarcopenia for each of the GT and GG genotypes of the rs2285679 (GC). Concerning to VDR SNP rs228570 the institutionalized seniors recognized with sarcopenia were 2 for AA, 3 for GA and 5 for GG (Table 3).

### 2.6. Comparisons between Single Nucleotide Polymorphisms vs. 25-Hydroxy Vitamin D

Table 4 shows the existence of significant differences (*p* < 0.05) in serum 25-OH/D levels between the different AA/GA/GG polymorphisms corresponding to SNP r2228570 (VDR) and TT/GT/GG belonging to rs2282679 (GC). In addition, statistically significant (*p* < 0.05) differences in 25-OH/D concentration were observed between patients carrying the GT and GG genotype with respect to TT of SNP rs2282679 (GC). Additionally, in r2228570 (VDR) statistically significant (*p* < 0.05) differences were obtained between the GA and GG genotypes with respect to the homozygous AA genotype (Table 4).

### 2.7. Correlations between the Concentration of 25-Hydroxy Vitamin D and Single Nucleotide Polymorphisms

Table 5 shows the correlations between total 25-OH/D concentration and the genotypes of the 3 SNPs included in the study. Weak or very weak positive correlations are observed with the AA (rs10741657), TT (rs2282679) and AA (rs228570) genotypes, being statistically significant (*p* < 0.05) in all three cases. However, moderate, and statistically significant (*p* < 0.05) negative correlations are obtained for the homozygous GG genotypes of rs10741657 (*r* = −0.32; *p* = 0.013), rs2282679 (*r* = −0.34; *p* = 0.011) and rs228570 (*r* = −0.46; *p* = 0.001) (Table 5).

### 2.8. Single Nucleotide Polymorphisms Associated with 25-Hydroxy Vitamin D Concentration

The results of multivariate logistic regression analysis yielded a diagnostic/predictor model of plasma 25-OH/D concentration consisted of three variables which were each bi-allelic variant of the SNPs that were included in the investigation rs10741657, rs2282679 and rs228570 (Table 6). The results of the multivariate analysis revealed that older adults with homozygous TT and AA were associated with higher 25-OH/D levels (>30 ng/mL) when compared with heterozygous GT and GA, respectively (rs2282679 [OR 1.23, 95% CI 0.78–1.76]; rs10741657 [OR 1.21, 95% CI 0.92–2.07]; and rs228570 [OR 1.19, 95% CI 0.82–1.88]). However, the homozygous GG was inversely associated with higher 25-OH/D levels (<30 ng/mL) when compared with homozygous GT and GA (rs2282679 [OR 0.88 95% CI 0.56–1.25]; rs10741657 [OR 0.84 95% CI 0.69–1.49]; and rs228570 [OR 0.64 95% CI 0.49–1.15]). None of these associations were statistically significant.

### 2.9. Correlation of Sarcopenia Degree and 25-Hydroxy Vitamin D (25-OH/D) Concentration

**Figure 1:**
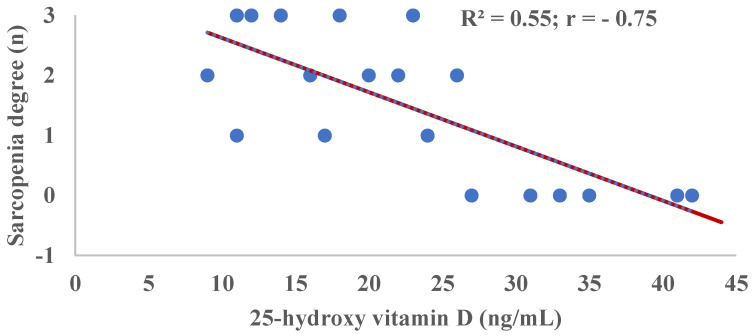
Correlation of Sarcopenia Degree and 25-hydroxy vitamin D (25-OH/D) concentration. Degrees of Sarcopenia: 0, absence; 1, Probable; 2, Confirmed; 3, Severe.

Figure 1 shows an R^2^ = 0.55 indicating that at least 55% of the variations in the degree of sarcopenia are responsible for the serological 25-OH/D concentration. In addition, a moderately negative correlation (r = −0.75) is shown between the degree of sarcopenia and 25-OH/D levels. The correlations of 25-OH/D with the biomarkers used in the identification of sarcopenia were: SMMI R^2^ = 0.56 r = 0.75; strength by Manual pressure dynamometry test R^2^ = 0.23 r = 0.48; physical performance evaluated by Get-Up-And-Go Test R^2^ = 0.47 r= −0.69 (results not shown in graphs or tables).

## 3. Discussion

Currently, health sciences research is trying to identify genetic factors and their implication in the development of multifactorial diseases to perform interventions from precision personalized medicine [32]. In this regard, genome assessment using SNPs would allow the detection of heritable factors such as mutations that contribute to increased disease risk [33]. In this study, we investigated the connection between CYP2R1 (rs10741657), GC (rs2282679) and VDR (rs2228570) on serum 25-OH/D concentrations and the relationship to the degree of sarcopenia in institutionalized elderly people. Our results show that 5 older adults were identified with sarcopenia for the GG genotype for each of the SNPs studied that also had a moderate negative and statistically significant correlation with respect to 25-OH/D levels. In addition, we observed significant differences in 25-OH/D concentration between the bi-allelic combinations of rs2282679 and rs2228570. We detected statistically significant weak positive correlations between the AA (rs10741657 and rs2228570) and TT (rs2282679) and 25-OH/D alleles. In addition, for these genotypes, homozygous TT and AA were associated with higher 25-OH/D levels (>30 ng/mL) when compared with heterozygous GT and GA, respectively, (rs2282679 and (rs228570). However, the homozygous GG was inversely associated with higher 25-OH/D levels (<30 ng/mL) when compared with homozygous GT and GA (rs2282679, rs10741657 and rs228570. None of these associations were statistically significant. We also show a strong negative correlation between the degree of sarcopenia and 25-OH/D levels.

Consistent with our study, two genes substantially affect vitamin D status are the GC gene and the CYP2R1 gene [34]. Variations in the GC and CYP2R1 genes are associated with an increased risk of having low plasma 25-OH/D levels [13]. Specifically, CYP2R1 (rs10741657) and GC (rs2282679) were significantly associated with vitamin D status. Thus patients with the GG genotype of CYP2R1 (rs10741657) and GC (rs2282679) were significantly more likely to have inadequate 25-OH/D levels [18]. Additionally, six alleles of two genes, GC and CYP2R1, were associated with a significantly lower vitamin D level in postmenopausal women, aged 50–79 years although the specific alleles were different [19].

The gene at the second locus, CYP2R1, encodes a liver microsomal enzyme described as the enzyme responsible for vitamin D hydroxylation in the liver [17]. From what is described in our results, which are supported by other studies [13,18,19,34], it could be hypothesized that CYP2R1 gene variants are related to adequate circulating 25-OH/D levels because it is the enzyme responsible for the first key step in vitamin D metabolism due to its influence on total 25-OH/D levels. GC encodes vitamin D-binding protein (DBP), a 52–59 kDA protein synthesized in the liver that binds vitamin D to its metabolites and transports them (25-OH D and activated vitamin D [1,25-OH/2D]) [17]. Some recent studies have reported significant associations between nonsynonymous GC as rs7041 (Asp → Glu) and rs4588 (Thr → Lys) with 25-OH D concentrations [13], although these nonsynonymous SNPs were not studied. Wang et al. [13] reported that the GG and GT bialeles, of the GC gene, were associated with lower 25-OH/D concentrations (like our results) were strongly associated with lower DBP levels. Decreased circulating DBP concentration influences the metabolism and availability of 1,25-OH/2D vitamin D to target organs, as well as the clearance of vitamin D metabolites from plasma. On the other hand, the decrease or loss of DBP function may be accompanied by changes in the relative proportions of free and bound 25-OH D, with the free fraction being the limiting factor in the production of 1,25-OH/2D [4].

The gene encoding VDR on chromosome 12q13.11 spans approximately 75 kb and contains 14 exons [20]. Among numerous (>60) SNPs identified in the VDR gene were linked to the risk of several disease such as autoimmune disorders, including rheumatoid arthritis, systemic lupus erythematosus, inflammatory bowel disease, diabetes mellitus, multiple sclerosis, an autoimmune disease, cancer or Parkinson [21,22,35,36]. However, these studies did not analyze the relationship between VDR polymorphisms and 25-OH D blood serum concentrations. The rs2228570 affects the translational initiation codon of the VDR, it is the only currently known polymorphism of this gene that results in altered protein expression and modifies its expression [37]. A single study [21] identified that VDR genotype variants (rs2228570), the same as in our research, are not risk factors for serum 25-OH/D depletion or vitamin D deficiency. Our results are contrary to those reported by Kamyshna et al. [21]. In fact, we found significant differences in blood levels of 25-OH/D and the different alleles of rs2228570. Moreover, the presence of AA would have a positive correlation and would be a protective factor for having optimal vitamin D levels and avoiding 25-OH D deficiency. The differences could be due to their study subjects being patients with thyroid pathology with suboptimal vitamin D levels (<20 ng/mL) at baseline. It should be considered that approximately more than 90% of Hashimoto’s patients were vitamin D deficient [38]. Therefore, the concentration of 25-OH/D would be mainly conditioned by thyroid disease and to a lesser extent by genetic determinants.

The results of this study demonstrate that GG allelic variations in CYP2R1 (rs10741657), GC (rs2282679) and VDR (rs2228570) genes affect a patient’s vitamin D sufficiency status that may increase the risk of sarcopenia. In fact, we found a moderately negative correlation (r = −0.75) is shown between the degree of sarcopenia and 25-OH/D levels. It is known that vitamin D is considered a key element in sarcopenia, where 25-OH/D concentrations are positively correlated with muscle mass, strength and physical performance in older adults [39]. These indicators were used to identify the different degrees of sarcopenia in our institutionalized elderly.

Vitamin D participates in skeletal muscle by modulating cell proliferation and differentiation. Optimal concentrations of 25-OH/D allow overexpression follistatin and insulin-like growth factor 2 (IGF-II) that induce more competent cell proliferation. The differentiation process is controlled by numerous myogenic transcription factors (fetal myosin, neural cell adhesion molecule, B-cell lymphoma 2, insulin-like growth factor-1, fibroblast growth factor, retinoblastoma protein and myogenic differentiation protein 1) that are induced by 25-OH/D [40]. Vitamin D has an impact on the diameter and quantity of type II muscle cells, especially type IIA [41]. This type of fast twitch muscle fibers is essential for the elderly because of their ability to reduce the risk of falls and facilitate the speed of execution of movements and changes of direction [42]. In older adults, the role of 25-OH/D that may be of most interest would be in muscle regeneration. Vitamin D drives the increase in cross-sectional area of skeletal muscle fibers by blocking the cell cycle [6]. Furthermore, vitamin D down-regulates the expression of myostatin (GDF-8) [7], a negative regulator of muscle, which prevents muscle degeneration, improves functionality of contractile elements, and increases muscle strength [43]. Vitamin D-VDR communication is considered as a key biological factor in skeletal muscle physiology. The proper vitamin D-VDR interaction in muscle satellite cells would suggest a key role of vitamin D in the development, muscle regeneration and repair of damaged musculoskeletal structures [20]. The importance of 25-OH/D on the muscular system in our patients because 56% of the variations in SMMI are the responsibility of the serological concentration of 25-OH/D establishing a strong and positive (r = 0.75) correlation between SMMI and 25-OH/D.

Vitamin D is associated with neuromuscular performance, showing a positive association of vitamin D levels with muscle strength and physical performance in the elderly [11]. This agrees with what was described in our study where in the strength test by manual pressure dynamometry test and physical performance assessed by the Get-Up-And-Go test. The influence of the blood concentration of 25-OH/D on skeletal muscle will be responsible for the effects on strength and physical performance [39]. In addition, the presence of VDR in myocytes should be considered, and that the lower vitamin D-VDR interaction evidenced a substantial decrease in reduced grip strength and exercise performance [44].

### Limitations and Strengths

Several limitations need to be acknowledged. First, the study included a limited number of participants, which limits the interpretation of the results. In addition, in this study patients were not asked to verify food intake, although the diet was similar, because the patients are subjected to the residence’s intakes programmed by a nutritionist.

Despite these limitations, the strengths of this study lie in the fact that the 25-OH/D levels shown in this study (Table 2) show similar figures for prevalence of vitamin D insufficiency/deficiency as in previous studies in the elderly population [45,46,47,48,49]. In addition, the participants did not receive vitamin D supplementation, which would not distort the 25-OH/D levels. Additionally, 20% of the samples were genotyped in duplicate as a quality control and all the results were congruent for 3 of the most important SNPs influencing plasma vitamin D concentration were studied. Thus, this might suggest that the analyses and results could be considered honest.

## 4. Material and Methods

### 4.1. Study Design

In total, 19 patients were included in this study. The patients were recruited from the Mixed Elderly Residence of Soria (Spain). The cohort consisted of older adults aged ≥ 75 years who were institutionalized elderly men not supplemented with vitamin D.

### 4.2. Data Collection

Two study investigators (D.F.-L. and J.L.G.H.) examined electronic medical records and performed specific tests designed for this study. Measures included in the data collection were sociodemographic and lifestyle; physical fitness and clinical characteristics (Table 1).

#### 4.2.1. Sociodemographic and Lifestyle

Gender, age, nationality (Spanish or other), body mass index (BMI), Skeletal Muscle Mass (SMM), Skeletal Muscle Mass Index (SMMI), sun exposure, tobacco consumption (smokers, ex-smokers, or never smokers), adherence to the Mediterranean diet, and self-perception of health status were included as sociodemographic and lifestyle characteristics. BMI was calculated according to Spanish Obesity Society (SEEDO) criteria [24], SMMI was evaluated by bioimpedance, and based on the bioelectrical parameters, body composition analysis was performed by applying age- and sex-specific prediction models using the Janssen equation [27], adherence to the Mediterranean diet was evaluated with a score using the 14-item questionnaire proposed by Trichopoulou et al. [25] and self-perception of health status was assessed employing a self-made visual analog scale adapted from Gould et al. [26].

#### 4.2.2. Physical Fitness

Manual pressure dynamometry was evaluated, in both hands, by performing two measurements with an electronic hand dynamometer (CAMRY MO. EH101, General ASDE, Madrid, Spain) starting with the dominant hand and with the arm in functional position, taking the highest value for each hand [28]. The risk of falling was evaluated through the “*Get Up and Go Test*”, a test proposed by Gálvez Cano et al. [29], in which the time required to get up from the chair, walk to a mark located 3 m away, turn around and sit back in the chair was considered, thus assessing the agility, balance, and resistance of the participants. When the participant’s time exceeded 30 s, a fall risk was considered [29].

#### 4.2.3. Clinical Characteristics

Data about clinical characteristics included as Presence of known drug allergies (yes/no); Previously passed COVID-19 infection (yes/no); Pathologies (arterial hypertension, obesity, insulin-dependent diabetes mellitus, respiratory, cancer, and cardiovascular); Usual treatment (antihypertensives, anticoagulants, immunosuppressants, anxiolytics or sedatives, hypolipidemic agents, antidiabetics and cardiovascular); Use of oxygen therapy (current, previous/occasional or never); vital signs (blood pressure [BP], heart rate [HR], temperature [T°] and oxygen saturation [O_2_] at rest were measured with Vital Signs Monitor RVS-100 (RIESTER, Jungingen Germany).

### 4.3. 25-OH/D Level Determination in Peripheral Blood

In December 2021, two blood samples, one for total serum 25-OH/D level and one for genetic testing, were obtained from each patient. EDTA plasma samples with sufficient volume were assayed for 25-OH/D concentration using a commercially available Enzyme-linked immunosorbent assay (ELISA) kit (Eagle Biosciences, Nashua, NH, USA). The 25-OH/D levels is the established biomarker of vitamin D status [50].

According to Spanish Society for Bone and Mineral Metabolism Research (SEIOMM) [31], which establishes 3 categories of based on 25-OH/D level for populations at risk of hypovitaminosis D in: (i) <10 ng/mL deficiency; (ii) Insufficiency 10–30 ng/mL; (iii) >30 ng/mL normal physiological range.

### 4.4. Sarcopenia Degree

Sarcopenia degree have been determined in relation to the criteria of the European Working Group on Sarcopenia in Older People (EWGSOP) [30]. Four grades have been established: No Sarcopenia (0°); Probable Sarcopenia (1°); Confirmed Sarcopenia (2°); Severe Sarcopenia (3°).

### 4.5. Single Nucleotide Polymorphism (SNPs) Selection

In total, we selected 3 genes related to vitamin D synthesis (CYP2R1), transport (GC) and vitamin D receptor (VDR). The SNPs of the genes were: rs10741657 to CYP2R1, rs2282679 to GC and rs2228570 to VDR. SNPs had a function already described in the literature based on their previous association with 25-OH/D and its related genotypes [18,21].

### 4.6. DNA Isolation and Genotyping

The second blood sample was placed in a BD Vacutainer^®^ ETDA anticoagulated test tube and stored in a refrigerated space at the practice site until the time of DNA extraction. Each test tube was labeled with a unique identification number that correlated with the patient’s individual consent form to keep patient information confidential throughout the data collection process. Genomic DNA was isolated from 20 mL of the blood sample using FlexiGene DNA Kit (Qiagen, Hilden, Germany). 3 SNPs (rs10741657 to CYP2R1, rs2282679 to GC and rs2228570 to VDR) were genotyped with KASPar assays (KBiosciences, Herts, United Kingdom and LGC Genomics, United Kingdom) according to manufacturer’s instructions. Overall, 20% of samples were genotyped in duplicate as quality control and all the results were congruent.

### 4.7. Data Management and Statistical Analysis

For descriptive analyses, means and standard deviations were calculated for continuous variables and frequencies and percentages for categorical variables. Comparisons between single nucleotide polymorphisms vs. 25-hydroxyvitamin D a general univariate linear test was performed comparing the gene variable and fixed factors SNPs and then a Bonferroni post hoc test was performed to determine differences between SNPs. Correlations were estimated with Spearman’s rank correlation coefficient for SNPs and 25-OH/D. Multivariate logistic regression models were constructed to explore the presence or absence of a characteristic or outcome according to the values of a set of predictors. Multivariate logistic regression models with corresponding odds ratio (OR) and 95% confidence interval (CI). We performed a crude and adjusted (for age and BMI) multivariate logistic regression to study the association between bi-allelic variants of each SNPs in the study and 25-OH/D concentration, defined as vitamin D concentration below or above 30 ng/mL. A two-tailed *p*-value < 0.05 was considered statistically significant. For 25-OH/D and the diagnostic parameters of sarcopenia and sarcopenia grades, regression models were performed and from the coefficient of determination (R^2^) the Pearson correlation coefficient (r) was calculated. A *p*-value < 0.05 was considered statistically significant. All analyses were performed using the STATA version 15 statistical package (STATA Corp., College Station, TX, USA).

### 4.8. Ethical Considerations

The study was approved by the local ethics committee of the Faculty of Health Sciences in the University of Valladolid (PI No. 20-1921). All subjects gave written informed consent in accordance with the Declaration of Helsinki.

## 5. Conclusions

The evidence reported in our study demonstrated that allelic variations in the CYP2R1 (rs10741657), GC (rs2282679) and VDR (rs2228570) gene affect the vitamin D sufficiency status of patients. Therefore, genetic determinants will be important factors on which 25-OH/D blood levels in our study have revealed that vitamin D levels are negatively correlated with the degree of sarcopenia in older adults.

## 6. Applications

The prevalence of hypovitaminosis D in the elderly is very high and is constantly increasing, generating an important social and economic problem that has a negative influence on the morbimortality and quality of life of the elderly [9,10]. Thus, it could be recommended to evaluate the plasma levels of 25-OH/D periodically in this population group to implement an early diagnosis and treatment. In addition, laboratory biomarkers should be included to allow assessment of current nutritional status and ongoing follow-up. In this regard, the practical application of the scientific knowledge gained in this research could allow institutionalized elderly people to use the results of genetic testing for personalized nutrition to monitor vitamin D status and avoid the health problems associated with its deficiency, especially on the modulation of sarcopenia. A genetic variant, such as an SNP, subordinates the 25-OH/D levels that condition the health status of the individual. Therefore, the genotype can be used to individualize dietary advice, although it is necessary to complement it with other patient characteristics such as sex, age, anthropometry, health status, family history, socioeconomic level, together with dietary and the presence of food intolerances or allergies. Dietary advice to combat hypovitaminosis D is mainly necessary to increase intake through food, but sometimes the diet is insufficient to meet the vitamin D needs of the elderly and as vitamin D production is lower [12], supplementation with individualized vitamin D preparations could be one of the most effective measures to counteract hypovitaminosis D.

## Figures and Tables

**Table 1:** Sociodemographic and Lifestyle, Physical Fitness, Clinical and Vital signs Characteristics-Related with study participants.

Characteristics	Full Cohort (*n* = 19)
**Sociodemographic and Lifestyle**	
Gender, *n* (%)	
Male	19 (100.0)
Female	0 (0.0)
Age (years), mean (SD)	82.9 (6.7)
Nationality, *n* (%)	
Spanish	17 (89.5)
Other	2 (10.5)
^1^ Body mass index (BMI), (kg/m^2^), mean (SD)	27.2 (5.2)
^2^ Skeletal Muscle Mass (SMM), (kg), mean (SD)	23.8 (6.8)
Skeletal Muscle Mass Index (SMMI) (kg/m^2^), mean (SD)	9.1 (2.6)
Sun exposure, (min·day^−1^)	17.37 (6.76)
<15 min·day^−1^, *n* (%)	5 (26.3)
> 15 min·day^−1^, *n* (%)	14 (73.6)
Smoker, *n* (%)	6 (31.5)
Non-Smoker	10 (52.6)
Never Smoker	3 (15.7)
^3^ Trichopoulou’s MedDiet score, mean (SD)	10.4 (1.9)
^4^ Self-perceived health status g (%), mean (SD)	72.9 (14.7)
**Physical Fitness**	
^5^ Manual pressure dynamometry (kg/cm^2^), mean (SD)	
Dominant hand	26.4 (5.6)
Non-dominant hand	19.6 (4.3)
^6^ Get-Up-And-Go Test (seconds), mean (SD)	15.6 (6.5)
Yes (<20 seg), *n* (%)	14 (73.6)
No (≥20 seg), *n* (%)	5 (26.3)
**Clinics**	
Known allergies, *n* (%)	
Yes	5 (26.3)
No	14 (73.6)
Previously passed COVID-19 infection, *n* (%)	
Yes	12 (63.15)
No	7 (36.8)
Pathologies, *n* (%)	
Arterial hypertension	17 (89.9)
Obesity	1 (5.2)
Insulin-dependent diabetes mellitus	2 (10.5)
^7^ Respiratory	2 (10.5)
Cancer	1 (5.2)
^8^ Cardiovascular	14 (73.6)
Usual treatment, *n* (%)	
Antihypertensives	17 (89.9)
Anticoagulants	3 (15.7)
Immunosuppressants	1 (5.2)
Anxiolytics/Sedatives	16 (84.2)
Lipid lowering agents	3 (15.7)
Antidiabetics	2 (10.5)
Cardiovascular	14 (73.6)
Use of oxygen therapy, *n* (%)	
Currently	1 (5.2)
Previous/Occasional	2 (10.5)
Never	16 (84.2)
**Vital signs, mean (SD)**	
Blood pressure	
SBP (mmHg)	127 (14.0)
DBT (mmHg)	72.3 (12.4)
Heart rate (bpm)	76.1 (12.7)
Temperature (°C)	35.8 (0.5)
Oxygen saturation (%)	98.9 (1.9)

*Abbreviations*: SD, standard deviation; kg, kilograms; m^2^, square meters; *n*, size; mmHg, millimeters of mercury; bpm, beats per minute; DBT, diastolic blood pressure; SBP, systolic blood pressure; °C, degrees Celsius; Values are expressed as mean (SD) for quantitative variables and as frequency (percentage) for categorical variables. ^1^ Results obtained according to Spanish Obesity Society (SEEDO) criteria [24]; ^2^ Assessed by Janssen et al. [27] after impedance analysis; ^3^ Score proposed by Trichopoulou et al. [25]; ^4^ Assessed by Visual Analogue Scale (VAS) adapted from Gould et al. [26]; ^5^ Dynamometer Measurements described by Bohannon [28]; ^6^ Fall risk measurement assessment using the “Get up and go” test proposed by Gálvez Cano et al. [29]; ^7^ Including respiratory failure, chronic obstructive pulmonary disease, asthma, and cystic fibrosis; ^8^ Including coronary heart disease, heart failure, venous and/or arterial insufficiency and stroke.

**Table 2:** Percentages of 25-hydroxy vitamin D characterized by ranges of deficiency, insufficiency, and normality.

Age (Years)	Sample (*n*)	25-OH/D (ng/mL) Mean (SD)	Deficiency *n* (%) <10 ng/mL	Insufficiency (%) 10–30 ng/mL	Normal (%) >30 ng/mL
<70	8	26.5 (11.8)	1 (5.2)	4 (50.0)	3 (37.5)
>70	11	19.5 (7.8)	-	9 (81.8)	2 (18.2)
82.9 (6.7)	19	22.7 (10.1)	1 (5.2)	13 (68.4)	5 (26.3)

Values are expressed as mean (SD) for quantitative variables and as frequency (percentage) for categorical variables. Characterization of 25-hydroxy vitamin D ranges in populations at risk of hypovitaminosis D by Spanish Society for Bone and Mineral Metabolism Research (SEIOMM) [31].

**Table 3:** Polymorphisms of genes, 25-hydroxy vitamin D concentration and degrees of sarcopenia of study participants.

Gen	SNPs	Allele	*n* (%)	Degrees of Sarcopenia (n°)				Sarcopenia
				Absence (0°)	Probable (1°)	Confirmed (2°)	Severe (3°)	Full Cohort (*n* = 19)
CYP2R1	rs10741657	AA	5 (26.3)	4	0	1	0	1
		GA	8 (42.1)	1	3	2	2	4
		GG	6 (31.5)	1	0	2	3	5
		AA/GA/GG	19 (100)	6	3	5	5	10
GC	rs2282679	TT	4 (21.1)	4	0	0	0	0
		GT	8 (42.2)	1	2	2	3	5
		GG	7 (36.8)	1	1	3	2	5
		TT/GT/GG	19 (100)	5	3	5	5	10
VDR	rs228570	AA	9 (47.3)	6	1	1	1	2
		GA	5 (26.3)	0	2	1	2	3
		GG	5 (26.3)	0	0	3	2	5
		AA/GA/GG	19 (100)	6	3	5	5	10

Values are expressed as frequency (percentage) for categorical variables. SNPs, Single nucleotide polymorphisms; 25-hydroxy vitamin D, 25-OH/D.

**Table 4:** Comparisons between single nucleotide polymorphisms vs. 25-hydroxy vitamin D.

Gen	SNPs	Alleles	25-OH/D (ng/mL), Mean (SD)	*p*-Value
CYP2R1	rs10741657	AA	30.0 (12.4)	0.084
		GA	22.7 (8.7)	
		GG	16.6 (5.9)	
GC	rs2282679	TT	37.7 (4.4)	<0.001
		GT *	20.5 (6.9)	
		GG *	16.7 (6.2)	
VDR	rs228570	AA	29.9 (9.5)	<0.001
		GA ^&^	18.6 (3.7)	
		GG ^&^	14.0 (5.1)	

Notes: Values are expressed as mean (SD) for quantitative variables. Statistically significant values at *p*-value level < 0.05. Multiple comparisons test is based on Bonferroni test. *: Significant differences respect to TT. ^&^: Significant differences respect to AA. SNPs, Single nucleotide polymorphisms; 25-hydroxy vitamin D, 25-hydroxy vitamin D.

**Table 5:** Correlations between the concentration of 25-hydroxy vitamin D and single nucleotide polymorphisms involved with vitamin D.

Gen (SNPs)	Full Cohort (*n* = 19)	
	*r*	*p*-Value
*CYP2R1 (rs10741657)*		
AA	0.18	0.035
GA	0.09	0.436
GG	−0.32	0.013
*GC (rs2282679)*		
TT	0.27	0.038
GT	0.08	0.541
GG	−0.34	0.011
*VDR (rs228570)*		
AA	0.16	0.031
GA	0.05	0.169
GG	−0.41	<0.001

Notes: Bold type equals statistically significant values at *p*-value level < 0.05. Correlations (r) are based on Spearman’s rank correlation coefficient. SNPs, Single nucleotide polymorphisms.

**Table 6:** Participant study characteristics and single nucleotide polymorphisms (SNPs) associated with 25-hydroxy vitamin D concentration. Crude and adjusted Multivariate logistic regression models with corresponding Odds Ratio (OR) and 95% Confidence Intervals (95% CI).

SNPs	Bi-Allelic Variant	Full Cohort (*n* = 19)	
		OR (IC 95%) Crude	OR (IC 95%) ^1^ Multivariate Analysis
*GC* *(rs2282679)*	GT	1.00 (ref.)	1.00 (ref.)
	TT	1.03 (0.56–1.74)	1.23 (0.78–1.76)
	GG	0.95 (0.74–1.17)	0.88 (0.56–1.25)
*CYP2R1 (rs10741657)*	GA	1.00 (ref.)	1.00 (ref.)
	AA	1.18 (0.63–1.89)	1.21 (0.92–2.07)
	GG	0.92 (0.54–1.17)	0.84 (0.69–1.49)
*VDR* *(rs228570)*	GA	1.00 (ref.)	1.00 (ref.)
	AA	1.10 (0.64–2.03)	1.19 (0.82–1.88)
	GG	0.78 (0.51–1.28)	0.64 (0.49–1.15)

*Abbreviations*: CI, confidence interval; BMI, body mass index; OR, Odds Ratio; ref, reference. Notes: ^1^ Multivariate Binary logistic regression model for diagnostic/predictor of plasma 25-OH/D concentration (defined as vitamin D concentration below or above 30ng/ml.) composed of rs10741657, rs2282679, and rs228570 (Single Nucleotide Polymorphisms (SNPs) had 3 bi-allelic variants) adjusted for age (continuous) and BMI (continuous)) from Table 6.

## Data Availability

Not applicable.
